# Nutritional Compounds to Improve Post-Exercise Recovery

**DOI:** 10.3390/nu14235069

**Published:** 2022-11-29

**Authors:** Emma O’Connor, Toby Mündel, Matthew J. Barnes

**Affiliations:** School of Sport, Exercise and Nutrition, Massey University, Palmerston North 4410, New Zealand

**Keywords:** exercise-induced muscle damage, EIMD, nutritional strategy, exercise recovery

## Abstract

The metabolic and mechanical stresses associated with muscle-fatiguing exercise result in perturbations to bodily tissues that lead to exercise-induced muscle damage (EIMD), a state of fatigue involving oxidative stress and inflammation that is accompanied by muscle weakness, pain and a reduced ability to perform subsequent training sessions or competitions. This review collates evidence from previous research on a wide range of nutritional compounds that have the potential to speed up post-exercise recovery. We show that of the numerous compounds investigated thus far, only two—tart cherry and omega-3 fatty acids—are supported by substantial research evidence. Further studies are required to clarify the potential effects of other compounds presented here, many of which have been used since ancient times to treat conditions associated with inflammation and disease.

## 1. Introduction

Over time, the performance of regular exercise induces adaptations that improve the functioning of the musculoskeletal and cardiovascular systems. Appropriately designed training programmes employ the core principles of overload and progression to induce adaptations through steady increases in training intensity and duration [1]. However, in the short-term, the metabolic and mechanical stresses associated with muscle-fatiguing exercise result in perturbations to bodily tissues that arise immediately after exercise and, depending on the level of damage induced, can persist for several weeks [2,3]. This state of fatigue, known as exercise-induced muscle damage (EIMD), is accompanied by muscle weakness, pain and a reduced ability to perform subsequent training sessions or competitions for a period of time [4].

## 2. Exercise-Induced Muscle Damage

The exact mechanisms responsible for EIMD are yet to be elucidated. However, it is commonly accepted that the response is biphasic. The primary EIMD phase is induced by high levels of mechanical loading and metabolic stress that disrupt the contractile and connective tissue components of skeletal muscle [4], leading to enhanced cell permeability. In addition, there is increased production of reactive oxidative and nitrogen species (RONS) during exercise due to the greater flux of oxygen through metabolic pathways, adding further stress to the muscular environment [5]. Other factors that may exacerbate muscle fatigue include exercise-induced depletion of fuel substrates (e.g., phosphocreatine and glycogen) and the accumulation of metabolic byproducts (e.g., ammonia and hydrogen ions), which alter the muscular and blood environments by, for example, increasing the level of acidity. Changes in metabolic properties may also induce afferent feedback from the exercising muscles, promoting an increase in central fatigue [6,7].

The structural and biochemical changes that occur during the primary EIMD phase trigger a secondary phase characterised by an inflammatory cascade. This phase is necessary for the clearance of damaged tissue and the initiation of tissue repair and adaptation. However, paradoxically, it is also associated with increases in inflammation and oxidative stress [4,6]. The major drivers of the inflammatory response are the white blood cells (WBC), particularly neutrophils, which are mobilised in response to the build-up of calcium ions in damaged muscle and the related protein damage and signalling effects. Neutrophils have been shown to accumulate in exercised muscle immediately after exercise, where they engulf and remove necrotic muscle tissue [4,8]. They are also associated with the release of pro-inflammatory cytokines, most notably TNF-ɑ, IL-1β and IL-6, which are believed to facilitate the repair of damaged tissue via messaging functions [4,6,9]. The cytokine response appears to be mediated by the stress hormones adrenaline, noradrenaline and cortisol as well as various growth hormones, which aid in the degradation of damaged muscle fibres [2]. However, despite their roles in muscle repair, the activity of stress hormones and pro-inflammatory cytokines exacerbates the oxidative state of the muscular environment and surrounding tissues, prompting further release of inflammatory compounds and RONS [2]. The accumulation of inflammatory compounds stimulates fluid build-up and nociceptor activation, both of which are associated with the feeling of pain [10,11].

From about 48 h post-exercise, there is increased movement of other leukocytes, namely the monocytes and macrophages, to the site of fibre damage. Like neutrophils, macrophages produce reactive species and cytotoxic enzymes that exacerbate muscle damage. However, it appears that once in damaged tissue, macrophages promote the proliferation of satellite cells, which act as precursors to skeletal muscle formation and are required for the repair of existing muscle fibres and the production of new ones [3]. Further, to aid in the restoration of homeostasis, macrophages induce an anti-inflammatory cytokine response, primarily involving the release of IL-10, IL-4 and IL-1 receptor agonist [9]. These cytokines inhibit the expression of pro-inflammatory cytokines, thereby controlling the magnitude of inflammation present and promoting the restoration of homeostasis [12].

Symptoms of the primary EIMD phase begin during exercise or in the immediate post-exercise period, while the secondary response comes into play at about 8–12 h after the cessation of exercise. A common response to EIMD is delayed onset muscle soreness (DOMS), a multi-day muscle pain response which peaks in intensity at 24–72 h post-exercise and can last for 5–7 days [13]. The severity and time course of EIMD symptoms and subsequent impacts on recovery and future performance depend on the type, intensity and duration of exercise performed and the individual’s level of habituation to that type of exercise [14]. High-intensity, long-duration and unaccustomed exercise modalities, including stepping exercise, very steep downhill running, very long distance running and single-joint maximal eccentric exercise, induce large amounts of mechanical and metabolic stress and are linked to excessive EIMD, while submaximal exercise and exercise involving primarily concentric (muscle shortening) contractions produce milder levels of damage [3,4]. Despite their low energy cost, eccentric (muscle-lengthening) muscular contractions induce significant EIMD (5) due to the greater mechanical stress they place on muscle fibres compared with other types of contractions [4]. This is likely because the high level of tension developed during the lengthening of a muscle results in a high mechanical load to fibre ratio, placing increased stress on individual muscle fibres [2]. The magnitude of damage resulting from eccentric exercise is greater when contractions are performed at a longer muscle length or with greater force or angular velocity [4]. 

## 3. Nutritional Strategies to Improve Post-Exercise Recovery 

Due to the exacerbation of muscle damage associated with the post-exercise inflammatory response, there has been significant research interest in the use of recovery strategies to reduce the production, or enhance the clearance, of compounds associated with inflammation and oxidative stress [2]. However, it is important to remember that the process of inflammation acts as a stimulus for adaptation from exercise training [15]. There is a growing body of research evidence showing the role of reactive oxygen and nitrogen species (RONS) as vital signalling molecules that are likely to be associated with blood vessel formation (angiogenesis), mitochondrial biogenesis, insulin sensitivity and skeletal muscle hypertrophy [16]. In addition, inflammatory molecules have key roles in muscle repair and remodelling [2]. Nevertheless, it is likely that there is a threshold beyond which muscle damage and repair have no further positive effects on adaptation. At this point, the level of adaptation may even be impaired due to a reduction in the individual’s ability to train [2]. It is this level of damage that recovery strategies aim to prevent.

Various dietary components contain nutritional compounds that have been shown, or have the potential, to speed up post-exercise recovery. In practical terms, the consumption of such compounds may improve performance during multi-day or multi-match sporting events or permit a greater training load to be undertaken with a decreased risk of injury and/or excessive fatigue development. Here, we present a range of compounds with varying levels of evidence regarding their effects on post-exercise recovery. Some of these compounds target the secondary phase of EIMD through antioxidant and anti-inflammatory actions, while others act to induce relaxation and reduce the perception of fatigue in the post-exercise period. The studies included in this review were sourced from the PubMed, Web of Science and Scopus databases using the following keywords and their combinations: “exercise”, “inflammation”, “recovery”, “muscle damage” and “nutrition”. Additional studies were sourced from the references of retrieved articles, where relevant. Searches were performed up to July 2022.

## 4. Compounds with Anti-Inflammatory Activity

The use of foods or supplements with antioxidant activity to enhance post-exercise recovery is a controversial area of research. The rationale for employing these products as part of a recovery strategy is their ability to directly interact with and neutralize oxidative species [17]. The heightened production of RONS during exercise and the subsequent inflammatory response surpass the ability of the endogenous antioxidant system to suppress the accumulation of these species, resulting in a state of oxidative stress, which is associated with muscle soreness, fatigue and reduced functionality, as well as an increased susceptibility to illness [18,19]. However, there is also concern that suppression of the oxidative response could attenuate physical adaptations to training, ultimately having a negative effect on future performance [19]. 

### Vitamins C and E

Vitamin C (L-ascorbic acid) is an important water-soluble vitamin with antioxidant characteristics that is mainly found in citrus fruits, with smaller quantities in sweet peppers, strawberries and cruciferous and leafy vegetables. Its primary activities are RONS neutralisation and the regeneration of other antioxidant molecules, such as vitamin E, β-carotene and glutathione [2]. Vitamin E (α-tocopherol) is a lipid-soluble, cell-membrane-based vitamin that can inhibit lipid peroxidation (the breakdown of fatty acids) and neutralise RONS. It is primarily found in vegetable oils, especially sunflower, safflower and nut oils [2]. Despite a large body of research on the effects of supplementation with vitamins C, E or a combination of the two, it is unclear whether these compounds affect post-exercise recovery positively, negatively or not at all, and it is likely that the effect is dependent on the population, mode of exercise and dose used.

In accordance with its known antioxidant activities, ingestion of vitamin C [20,21], the dominant antioxidant in plasma [20], has been associated with an improved serum antioxidant status. Numerous studies have also recorded reductions in markers of oxidative stress [20,21,22] and muscle damage [20,23,24,25] following muscle-fatiguing exercise modalities such as downhill running, maximal resistance exercise and endurance exercise protocols. Exceptions to this have occurred in cases where an acute dose prior to exercise was employed rather than a daily dose over multiple weeks [26], or when the vitamin C dose given was less than 1000 mg/d ([26,27,28,29]. In terms of the immune response, evidence is varied, with both a decrease [22,26,30] and a lack of change in response [21,27,29] to muscle-fatiguing exercise by inflammatory markers recorded following vitamin C supplementation.

While there is evidence that supplementation with vitamin C can reduce blood markers of damage during the post-exercise response, it is less clear whether this benefits physical recovery. Supplementation with 1000 mg/d vitamin C for multiple weeks was shown to improve muscle force recovery and reduce post-exercise muscle soreness in untrained males following a 90 min intermittent run test [26]. However, another study employing a similar supplementation protocol found no differences in measures of muscle force recovery or function following eccentric elbow exercise [24]. It is possible that the variation in results between these two studies could have been influenced by the different exercise modalities used. Interestingly, when multi-day supplementation of vitamin C was employed primarily in the post- rather than the pre-exercise period, muscle function decreased despite a reduction in markers of oxidative stress [23]. Similarly, an acute vitamin C supplementation protocol was associated with reduced function in some muscle groups following an intermittent run test, compared with a placebo condition [26]. 

In contrast to the large body of research on vitamin C, the use of vitamin E supplementation to improve post-exercise recovery has received less attention. Jakemani et al. [31] found no effect of supplementation with vitamin E on muscle function, blood markers of muscle damage or antioxidant status following intense stepping exercise. Further, Avery et al. [32] associated the consumption of a high dose of vitamin E (1200 IU) for 12 days with increased muscle damage. However, under hypoxic conditions, equivalent to those present at an altitude of 4200 m, an environment associated with greater RONS production, vitamin E supplementation reduced blood markers of muscle damage and decreased markers of inflammation [33]. 

Since vitamin C interacts with vitamin E during the neutralisation of oxidative species, it was proposed that the combination of vitamins C and E could have a greater effect on post-exercise recovery than supplementation with either vitamin alone, but studies involving this combination have generally not yielded favourable results. Yfanti et al. [34] found that 16 weeks of combined vitamin C and E supplementation was associated with increased inflammation despite an improved antioxidant status, and Theodorou et al. [35] showed no effect of supplementation on muscle soreness, blood markers of muscle damage or antioxidant status. However, the consumption of 500 mg vitamin C and 400 IU vitamin E for 16 weeks did not reduce adaptation to an endurance training programme [18].

While there has been a significant focus on the potential of vitamins C and E to enhance post-exercise recovery, there is little evidence that these compounds are associated with improvements in physical parameters, despite the ability of vitamin C to enhance the antioxidant status and reduce blood markers of muscle damage. Since these vitamins act directly on reactive species, it has been suggested that they may even have a negative effect on adaptation to training by blocking signals to adaptive pathways. However, a recent meta-analysis indicated that there is little evidence that this is the case [19]. Overall, there is only moderate evidence that vitamins C and E may benefit recovery.

## 5. Fruit-Derived Polyphenols

Polyphenols are a group of phytochemicals with common structural characteristics that are found in high concentrations in a wide range of fruits and vegetables, strongly influencing their taste and pigment properties [36]. There are four main classes of polyphenols—lignans, phenolic acids, stilbenes and flavonoids—each encompassing many sub-classes [6]. In plants, polyphenols play roles in numerous critical processes, including growth, pollination and resistance to pathogens and environmental stressors [6]. 

Rationale for the use of polyphenols to improve post-exercise recovery in humans is based on their (1) signalling activities that upregulate the endogenous antioxidant response; (2) protective effects on red blood cells; and (3) enhancement of vascular function. Polyphenols also contain structural elements that can act as RONS scavengers [37], but they appear to mostly exert their antioxidant effects indirectly [6]. 

There is a large body of evidence linking polyphenol consumption with the suppression of oxidative stress and inflammation [6,38]. The results of previous research indicate that the daily ingestion of polyphenols for at least 3 days prior to and following an intense exercise session may enhance recovery from muscle damage. However, the effectiveness of polyphenol-containing supplements varies depending on their source. A key factor to consider is the influence of the growing conditions and post-harvest processing on the final polyphenol composition of a particular batch of supplements. Since the reporting of such conditions is rare in studies published on polyphenols and exercise recovery, the optimal conditions are unknown [6]. 

### 5.1. Tart Cherry

Montmorency tart cherry (TC), which can be consumed as a beverage made from powdered extract or liquid concentrate, contains polyphenols primarily from the anthocyanin (flavonoid) subclass. It has been associated with improved post-exercise muscle force and functional recovery (performance in a jump or agility task) across a variety of exercise types, including resistance-based [39,40], repeated sprint [41,42] and endurance exercise [43,44] tasks, and in both trained [39,41,42,44,45,46] and untrained/recreationally active [40] participants. This has typically been accompanied by a decrease in post-exercise muscle soreness [40,41,42,45,46]. 

Alongside physical indicators of improved recovery, numerous studies have shown an attenuation of the post-exercise inflammatory response following TC supplementation [41,43,44,46]. This effect appears to only involve particular components of the immune cascade, namely those involved in the initial stage [6]. 

Previous research indicates that, to improve recovery, a TC drink containing ~600 mg polyphenols (equivalent to about 30 mL concentrate) should be consumed twice daily for at least 3 days prior to exercise [6]. Studies employing lower doses of TC have generally shown a lesser effect on post-exercise recovery [45]. Overall, it appears that TC has the potential to be used by athletes to attenuate the inflammatory response and speed up recovery post-exercise. Despite the lack of findings regarding inflammatory markers in studies employing resistance-based exercise protocols, the improvement in muscle function across both endurance and resistance exercise modalities indicates that TC can be considered to improve recovery across all types of exercise. 

### 5.2. Pomegranate

Pomegranate (POM) contains polyphenols (~3.8 mg·mL^−1^ in POM juice) primarily from the ellagitannin subclass (80–90%) with smaller amounts of anthocyanins (8–15%) [47]. It was used historically to treat a variety of inflammatory conditions [48] and has been linked to a decline in cancer proliferation [49], the amelioration of cardiovascular disease markers [50] and decreases in gut and joint inflammation [51,52]. POM has also been shown to improve the blood antioxidant status and reduce markers of oxidative stress following resistance-based exercise when consumed multiple times in the 48 h prior to exercise [53], suggesting that it may have an effect on post-exercise recovery.

The effects of POM consumption on post-exercise recovery have only been investigated in relation to resistance-based exercise, and varying results have been obtained. In untrained males, the consumption of POM juice for at least 3 days prior to a maximal resistance-based exercise task was associated with improved post-exercise force recovery in both the elbow [54] and knee [55,56] muscles, despite no apparent effects on muscle soreness. In contrast, in trained males, reduced post-exercise soreness and improved force recovery were found following elbow but not knee exercise [55], suggesting that the knee exercise protocol used did not induce sufficient muscle damage in these trained individuals to allow an effect of POM to be observed. Only one study has measured blood markers of inflammation, but since the exercise task used in that study failed to induce a large inflammatory response, no effect of POM could be observed [54].

In addition, the consumption of POM prior to exercise has been associated with increases in post-exercise blood vessel diameter and blood flow [57,58], suggesting a link between pomegranate consumption and oxygen delivery and providing a further potential mechanism for improved recovery. However, the effects of these changes on post-exercise recovery have not yet been investigated.

The dose of POM associated with improvements in post-exercise recovery is 500 mL of POM juice or 30 mL of POM concentrate providing ~650 mg of polyphenols, consumed daily for at least 5 days prior to exercise. Blood flow changes have been recorded with an acute dose of 1000 mg of POM extract (3500 µmol/L polyphenols), 30 min prior to exercise [57,58]. Overall, the literature suggests that the consumption of POM juice may speed up recovery from resistance-based exercise, but its effects following endurance exercise are unknown, and the size of the effect may be dependent on the individual’s training status.

### 5.3. Blueberries

Among the common berry fruits, blueberries are especially high in polyphenols from the anthocyanin subclass [59]. Previous research has provided evidence that the consumption of blueberries, either as fresh/frozen fruit or in freeze-dried powder form, may improve strength recovery following resistance-based exercise [60] or enhance muscle function following exhaustive endurance exercise [61].

However, in general, blueberry consumption has not been associated with declines in blood indicators of muscle damage or inflammation [60,62,63], aside from in one study that recorded a drop in a key pro-inflammatory compound (IL-6) in a group of sedentary adults who performed treadmill walking [64]. 

There is some evidence that polyphenols may influence blood flow through their inhibitory effect on enzymes associated with the bioavailability of nitric oxide, a key controller of blood vessel dilation [57]. A polyphenol-induced increase in blood flow could accelerate lactate clearance, enhancing the rate of post-exercise recovery. Accordingly, in a group of trained runners, a decrease in the blood lactate concentration was recorded following an 8 km all-out running time trial when blueberry was consumed for 4 days prior to exercise [61]. 

Regarding the dose of blueberry required to produce positive effects, a wide range of supplementation protocols have been employed, with durations ranging from 72 h to 8 weeks prior to exercise [60,61,62,63,64]. The minimum dose given to produce a positive effect on physical recovery was 600 mg (1360 mg polyphenols), spread over three drinks, on the day of exercise and 200 mg (420 mg polyphenols) per day for two days post-exercise [60]. 

Overall, while there is some evidence that blueberry may positively affect post-exercise recovery, research on this compound is limited and further studies are needed to corroborate existing results.

### 5.4. Blackcurrant

Like blueberry, blackcurrant is rich in polyphenols from the anthocyanin subclass (130–460 mg/100 g fruit depending on the source), with some research claiming that the type of anthocyanins present in blackcurrant (delphinidin glycosides) has more effective antioxidant activity than that present in blueberries (malvidin glycosides) [15].

When consumed as either an extract or juice, blackcurrant has been associated with an improved ability to resolve the acute inflammatory response post-exercise [65,66,67,68]. There is also some evidence that blackcurrant intake reduces exercise-induced muscle damage, as shown by post-exercise reductions in blood markers of damage [65,66,67,69], faster recovery of muscle function and lower ratings of perceived pain [69] after supplementation. 

The dose required to induce changes in the inflammatory response appears to be a minimum of 3.2 mg/kg (providing ~80 mg anthocyanins). The consumption of blackcurrant extract has been associated with an increase in the plasma anthocyanin content after 30 min, with the concentration peaking at 2 h [66]. Thus, it is recommended that blackcurrant should be consumed 1–2 h prior to exercise to take advantage of this time window [15]. Like blueberry, despite its potential to improve post-exercise recovery, there is a lack of research on blackcurrant.

### 5.5. Curcumin 

Curcumin, the polyphenolic compound responsible for the yellow colour of turmeric spice, has antibacterial, anti-inflammatory, antioxidant, wound healing and glucose-lowering properties [70]. Due to its regulatory effects on the inflammatory response, curcumin may improve post-exercise recovery. Although curcumin is well-tolerated in humans and is recognized as safe by the United States Food and Drug Administration (FDA), it is poorly absorbed in the gastrointestinal system and, when consumed in its natural form, is primarily excreted [71,72]. However, numerous delivery systems have been designed and demonstrated to improve its bioavailability. Components of these systems include piperine (the active component of black pepper), adjuvants, nanoparticles, liposomes, micelles, and phospholipid complexes [71]. All studies involving human participants presented in this review employed one of these systems. 

An initial study on the effects of curcumin in mice demonstrated improvements in voluntary activity and running performance following a muscle-damaging exercise protocol as well as reductions in blood indicators of muscle damage and inflammation [73]. Subsequently, a large number of studies involving human participants have shown similar positive effects. Improvements in muscle force recovery and/or function were shown following downhill running [72] and resistance-based exercise [74,75,76] when curcuminoids were consumed acutely [74] prior to exercise or in multi-day protocols [72,75,76]. A further study conducted in elite male rugby players showed an improvement in one-legged sprint performance but not countermovement jump height or muscle force recovery following a downhill one-legged hop protocol with multi-day curcumin supplementation pre- and post-exercise [77]. This may reflect the highly trained nature of the participants who may have had a greater endogenous antioxidant response and therefore received less benefit from the supplement [71]. 

Acute and multi-day curcumin supplementation protocols have also been associated with decreases in muscle soreness [74,75,78,79,80] and blood markers of damage [75,76,77,78,79,80,81] following muscle-damaging exercise. In line with this, curcumin supplementation has been linked with improvements in markers of antioxidant activity [78,82] and reductions in oxidative stress [82]. Lastly, the attenuation of multiple components of the inflammatory response has been observed in some studies [80,81]. However, others have shown no differences in inflammatory compound concentrations compared to placebo conditions [75,76], and one study noted increases in inflammatory compounds, which were associated with a reduced thigh circumference (indicating reduced swelling), following curcumin supplementation. The authors postulated that this indicated an improved immune response, resulting in a quicker return to baseline muscle status [74]. Thus, while curcumin appears to influence the immune response, the exact nature of this influence is unclear.

Improvements in elements associated with post-exercise recovery have been shown with a range of curcumin doses and supplementation protocols. McFarlin et al. [81] compared the effects of three doses of bioavailable curcumin (200, 400 and 1000 mg/day) consumed for 2 days pre- and 4 days post-exercise on TNF-ɑ, an inflammatory compound, following muscle-damaging exercise and showed that 400 mg/day was the lowest dose that resulted in its inhibition. It is unclear whether acute or multi-day curcumin supplements protocols are more effective for improving post-exercise recovery, and Takahashi et al. [82] showed improvements in markers of oxidative stress and antioxidant status following both acute (pre-exercise) supplementation and two-dose (pre- and post-exercise) protocols. It is important to note that, in rare cases, products containing turmeric have been associated with hepatotoxicity [83]. However, an extensive review of research on the safety of turmeric and curcumin consumption concluded that, even at a high dose of 6000 mg/day for 4–7 weeks, oral consumption of curcumin is not associated with toxicity [84]. Overall, curcumin appears to be a promising supplement for improving post-exercise recovery, although the most effective supplementation protocol is yet to be determined.

### 5.6. Beetroot Juice

Beetroot contains a number of bioactive compounds, including phenolic compounds, ascorbic acid, carotenoids, flavonoids and betalain pigments, the group of antioxidant compounds responsible for its violet colour, which have been shown to attenuate oxidative stress-induced injury and upregulate endogenous antioxidant production [85]. In addition, beetroot consumption has been associated with improvements in oxygen use and performance during submaximal endurance and resistance exercise in low to moderately-trained athletes, a phenomenon that has been attributed to its high nitrate content [86,87,88,89,90]. However, whether BJ can also invoke improvements in post-exercise recovery is under debate.

When consumed in multiple doses of 150–250 mL over several days (either post-exercise only or pre- and post-exercise) following a simulated soccer match [91] or repeated sprint exercise protocol [85], BJ was associated with improvements in post-exercise muscle function and reduced muscle soreness despite no changes in blood markers of muscle fatigue and oxidative stress. However, no improvements in muscle function or soreness or changes in blood markers of muscle damage, oxidative stress or inflammation were observed with similar BJ supplementation protocols following endurance running or cycling or an eccentric ankle exercise protocol [92,93,94,95]. Interestingly, Clifford et al. [95] showed that the consumption of BJ did not blunt the repeated bout effect, whereby the completion of unaccustomed eccentric exercise confers a protective effect in subsequent exercise bouts of that type for about 24 weeks [96]. This is significant because it suggests that BJ consumption does not negatively affect post-exercise adaptation. 

In terms of an appropriate supplementation quantity, since the majority of research in this area has been conducted by the same research group, a similar protocol has been used in most studies: 7–8 doses of ~250 mL BJ, containing 400 mg phenolic compounds, 194 mg betanin and 210 mg nitrate, consumed over 3–4 days [85,92,95]. This protocol has produced mixed effects on exercise recovery but has not been associated with adverse effects. 

## 6. Proteins and Amino Acids

### 6.1. Branched Chain Amino Acids

The branched chain amino acids (BCAAs), leucine, isoleucine and valine, are essential amino acids characterized by the presence of a branched-chain residue in their structure that are transported and metabolized similarly in the body. BCAAs are nutritional components of interest in research on post-exercise recovery due to their importance in protein metabolism [97]. They can be used as a fuel source when carbohydrate stores are exhausted after prolonged exercise, thereby suppressing post-exercise use of muscle protein for energy production and enhancing muscle regeneration [98]. Accordingly, supplementation with BCAAs, either acutely or for 12 days pre-exercise, has been linked with post-exercise reductions in blood indicators of muscle damage, such as CK, ammonia, lactate dehydrogenase (LDH) and myoglobin [99,100,101,102]. These changes have been associated with enhanced post-exercise muscle function recovery and decreased muscle soreness following repeated drop jumps [100] and maximal resistance-based exercise tasks [102,103,104]. 

BCAA supplementation may also reduce central fatigue in the brain by competing with free tryptophan, a precursor to the fatigue-inducing compound serotonin, for transport across the blood–brain barrier (BBB). By occupying transport carriers on the BBB, BCAA may reduce the amount of serotonin produced in the brain, thereby reducing or delaying fatigue during exercise [98]. In line with this theory, Kim et al. [101] recorded a reduction in post-exercise serotonin following exhaustive cycling exercise when BCAAs were consumed acutely 50 min prior to exercise.

In a review of the literature on BCAA supplementation [105], it was recommended that, for maximal benefit, supplementation with BCAA should occur for at least 1 week prior to exercise with additional doses on the day of exercise and supplementation on follow-up days. However, changes in markers of muscle damage have also been seen with relatively low BCAA doses of ~5.4–8.3 g consumed acutely prior to exercise [101,104]. Typically, BCAA supplements are made up of leucine, isoleucine and valine in a 2:1:1 ratio [100,101,104,106]. However, based on research associating the leucine component with changes in inflammatory processes in skeletal muscle [107], and given the reported competition between leucine, isoleucine and valine for cellular transport and metabolic processes, it has been suggested that supplementation with leucine alone may be sufficient to provide benefits to post-exercise recovery [108]. 

### 6.2. β-Hydroxy β-Methylbutyrate

β-Hydroxy β-methylbutyrate (HMB), a metabolite of the BCAA leucine, is a key modulator of protein synthesis and muscle repair. Among its proposed effects are the stimulation of pathways associated with growth hormone production, enhanced tissue repair through increased proliferation of satellite cells (muscle stem cells), improvements in muscle contractility and factors related to aerobic energy production, delayed muscle fatigue and greater immune function [109]. 

Nissen [110] was the first to associate the intake of HMB with muscle preservation, showing a decrease in muscle breakdown during the first 2 weeks of a resistance-training programme and a reduction in blood markers of muscle damage after a third week of training. This indicates that this supplement could enhance post-exercise recovery by reducing the level of damage incurred. In subsequent studies, daily HMB consumption during multi-week resistance training protocols was associated with reductions in markers of muscle fatigue as well as greater training adaptations [111,112]. 

Regarding the effect of HMB supplementation on recovery from a bout of muscle-fatiguing exercise, Knitter [113] associated 6 weeks of HMB intake with reduced inflammation following a 20 km cross country running race, and Nunan [114] showed an improvement in muscle function following downhill running when HMB was consumed for 11 days pre- and 3 days post-exercise, despite no changes in muscle soreness or markers of muscle fatigue. Further, Hoffman [115] linked 23 days of daily HMB supplementation by soldiers with preserved muscle quality and reductions in markers of the inflammatory response following an extreme physical training challenge involving walking with heavy packs while sleep deprived. 

HMB supplementation has also shown positive effects on recovery when consumed daily for shorter periods of time or acutely prior to or following exercise. Townsend [116] associated the intake of HMB for three days following muscle-fatiguing exercise with decreased markers of the inflammatory response in resistance-trained males. In addition, Wilson and colleagues found that acute HMB intake 30 min prior to eccentric lower leg exercise led to decreases in markers of muscle protein breakdown [117] and trends toward reductions in markers of muscle damage and improved muscle force recovery [118]. In addition, Arazi [119] showed reductions in markers of muscle damage following a plyometric protocol with just a low dose of HMB (1 g) taken 30 min prior to exercise.

However, although there is a significant body of research associating HMB intake with improvements in markers of post-exercise recovery, these findings have not been universal. A collection of studies involving various acute [120,121] and short-term [122,123] HMB supplementation protocols have failed to show differences in markers of inflammation [121,123] or muscle damage [121,122], breakdown [122] or soreness [121,123] following muscle-fatiguing exercise, suggesting that acute/short term supplementation protocols may not always be sufficient to induce beneficial effects on post-exercise recovery. 

Regarding the appropriate dose of HMB, the consumption of 3 g/day, split into 3 doses, for at least 2 weeks [110,112,113,114,115] has generally been associated with improvements in markers of recovery, while lower doses or shorter protocols have produced varied results. HMB can be consumed as a calcium salt (Ca-HMB) or in a free acid gel form (HMB-FA) with the HMB-FA form having greater bioavailability and inducing a faster increase in the plasma HMB concentration [124].

### 6.3. Creatine Monohydrate

Creatine, an amino acid that is highly expressed in skeletal muscle, is an essential contributor to energy production during short bouts of high-intensity exercise through the ATP–phosphocreatine (PCr) shuttle [125]. Daily intake of ~20 g of creatine monohydrate (Cr) for at least 2 days has been shown to increase the PCr concentration in human muscle by up to 50% [126]. There is substantial evidence that Cr can act as an ergogenic aid, producing improvements in power production [127], strength [128], anerobic threshold [129], aerobic work capacity [129,130] and repeated sprint performance [131,132,133,134] as well as an increase in muscle mass [133]. 

Cr has also been proposed for use as a recovery aid, particularly in cases where large amounts of muscle damage have occurred [125]. In addition to its role in energy production, it has been suggested to play roles in a number of recovery-related process including protein synthesis, muscle membrane stabilization, the regulation of calcium homeostasis, glucose storage, antioxidant activity and modulation of the post-exercise inflammatory cascade [17,125]. Accordingly, Cr supplementation has been associated with enhanced muscle glycogen supercompensation following exhaustive cycling exercise [135] and a reduction in the accumulation of blood markers of muscle damage after an ironman triathlon [136] and a 30 km running race [137].

However, results regarding recovery from resistance-based exercise have been varied. In an early study, Willoughby and Rosene [138] associated 12 weeks of Cr supplementation (6 g/day) with a reduction in perceived muscle soreness following unaccustomed resistance exercise. Subsequently, Cooke [139] found that the ingestion of Cr (0.3 g/kg BW/day) during a 5-day loading period prior to an eccentric exercise workout followed by a maintenance dose (0.1 g/kg BW/day) for 14 days post-exercise attenuated the post-exercise increase in blood markers of muscle damage and improved muscle force recovery. In addition, Veggi [140] showed that 6 days of Cr supplementation (20 g/day) prior to eccentric bicep exercise attenuated the post-exercise rise in blood markers of muscle damage, decreased muscle soreness and prevented a post-exercise drop in joint range of motion, indicative of improved muscle function.

In contrast, several other studies have shown no positive effects of similar Cr supplementation protocols on markers of muscle damage or physical recovery following resistance-based exercise [141,142,143]. Interestingly, Rosene [144] found no effect of Cr supplementation on post-exercise recovery when consumed in a dose of 20 g/day for 7 days pre-exercise. However, when supplementation was continued with a maintenance dose of 6 g/day for an additional 23 days, muscle force recovery following an eccentric knee extension protocol improved, despite no difference in blood markers of muscle damage. Thus, it is possible that an extended supplementation protocol may produce better recovery outcomes. It has also been suggested that the response to Cr supplementation differs between individuals and there are responders and non-responders to its use [125]. 

In general, it appears that for responders to Cr supplementation, a dose of ~20 g/day for 8–10 days, encompassing the pre- and post-exercise periods, is sufficient to reduce exercise-induced muscle damage and improve physical recovery. However, for some individuals, a longer supplementation protocol of several weeks may be more appropriate. Despite the proposed benefit of Cr on the inflammatory response, research evidence in this area is lacking, and the exact mechanism by which Cr may exert positive effects on post-exercise recovery is unknown. 

### 6.4. l-Glutamine

Glutamine is an amino acid that is abundant in human muscle and plasma. It plays regulatory roles in numerous cell types, acts as a fuel source for intestinal and white blood cells and is essential for the correct functioning of the inflammatory response [145,146]. Under resting conditions, endogenous production of glutamine in the skeletal muscles is sufficient to fulfill these roles, but under conditions of stress (e.g., due to muscle-damaging exercise), intramuscular and plasma concentrations of glutamine decrease, which may impair immune system function [145]. Thus, it has been suggested that supplementation with l-glutamine, the bioavailable form of glutamine, could be used to restore glutamine homeostasis and improve post-exercise recovery [146]. 

Two studies showed a reduction in post-exercise muscle soreness in untrained individuals who completed a multi-day glutamine supplementation protocol (0.3 g/kg bodyweight (BW)/day [145,146]. However, while an improvement in muscle force recovery was observed in male participants [145,146], one study noted that the same effect did not occur in females [145]. 

Regarding dose, an l-glutamine dose of 0.3 g/kg BW/day was not sufficient to alter the response of blood markers of muscle damage [146]. However, this was achieved with a much higher dose of 1.5 g/kg BW/day for 7 days [147]. In addition, a dose of 0.1 g/kg BW/day for 4 weeks was insufficient to induce any changes in post-exercise recovery [148]. Despite the rationale for glutamine supplementation being its role in the inflammatory response, no studies on exercise recovery have measured markers of inflammation following l-glutamine supplementation, although improvements in immune function have been noted in non-exercise-related research [149,150]. 

Overall, there is some evidence that l-glutamine has the potential to enhance post-exercise recovery in untrained individuals. However, its effect on trained athletes, whose immune response characteristics may differ from those of untrained individuals, is unknown. Thus, further research on the effects of l-glutamine supplementation in different populations is required.

### 6.5. l-Carnitine

l-Carnitine, an amino acid derivative that is primarily stored in the skeletal and cardiac muscles [151], plays an essential role in fatty acid oxidation by facilitating the transport of acetyl groups into the mitochondria to allow energy production through beta-oxidation. In addition, l-carnitine has been demonstrated to improve the antioxidant status and reduce oxidative stress [151]. Endogenous synthesis of l-carnitine provides about 25% of a human’s daily requirement for the compound, and the remainder must be ingested in the diet, with red meat being the richest source [152]. Under stressful conditions, the body has a greater demand for l-carnitine. Thus, supplementation with this compound may enhance recovery by preventing a post-exercise deficiency and thereby maintaining blood flow and oxygen supply, reducing structural and biochemical muscle damage and facilitating tissue repair [152].

As a supplement, l-carnitine is generally ingested as l-carnitine l-tartrate (LCLT) to increase its bioavailability and speed up its absorption [153]. Ingestion of LCLT for 2–3 weeks prior to a muscle-damaging exercise bout has been associated with reduced muscle soreness in the post-exercise recovery period in trained and untrained, young [151,154] and older (45–60 years) [155,156] adults with corresponding attenuation of multiple markers of muscle damage. The ability of l-carnitine supplementation to enhance muscle function in the post-exercise period has varied between studies. However, this may have been due to the use of inappropriate tests (mobility and hand grip exercises) to assess muscle function [154,155]. A recent study that assessed countermovement and squat jump performance in the days following a muscle-fatiguing lower body resistance-based exercise challenge recorded improvements in muscle function when a 5-week l-carnitine supplementation protocol was used [151].

The dose of LCLT associated with reduced muscle damage and improved post-exercise recovery is 2 g/kg BW/day for 2–5 weeks prior to exercise [151,154,155,156,157,158]. LCLT consumption could be particularly effective for non-meat-eaters, who may struggle to ingest sufficient l-carnitine through dietary sources. Interestingly, during the menstruation phase, females have decreased levels of serum carnitine, but supplementation with l-carnitine can attenuate this deficiency, potentially improving both exercise performance and recovery [151].

### 6.6. Watermelon Juice (l-Citrulline)

Watermelon juice (WJ) contains a number of bioavailable compounds with the potential to aid in post-exercise recovery, most notably l-citrulline, lycopene, glutathione and vitamins A and C. l-citrulline, a product of l-arginine oxidation, is of particular interest as it plays an essential role in the removal of ammonia, a metabolic waste product associated with fatigue, in the form of urea [159,160]. While powdered citrulline malate does not appear to enhance post-exercise recovery [161,162,163], WJ contains l-citrulline in a more bioactive form and may be an effective dietary intervention [164]. In addition to l-citrulline, the lycopene, glutathione and vitamins in watermelon juice could enhance post-exercise recovery through their antioxidant and anti-inflammatory properties [165]. 

Despite the potential of WJ to act as a recovery aid, research on its effects on post-exercise physical recovery and inflammatory markers is lacking. In resistance-trained males, the consumption of WJ enriched with l-citrulline and ellagitannin polyphenols prior to resistance-based exercise was associated with improved muscle force recovery and reduced muscle soreness. However, these effects were not observed with natural WJ [166]. In contrast, in untrained males, the consumption of both WJ and l-citrulline-enriched WJ reduced muscle soreness and improved heart rate recovery following short cycling efforts [167]. Shanely et al. [165] associated WJ consumption with increases in markers of the antioxidant status, but unlike other antioxidant-containing foods, WJ has generally not been associated with changes in blood markers of muscle damage [166], fatigue [164,165,167] or inflammation [165] post-exercise.

Thus, it remains unclear as to whether WJ can enhance post-exercise recovery, and further research is required to determine what dose, if any, could be used in this regard. In addition, mild gastrointestinal discomfort has been reported following the ingestion of large doses of WJ, possibly due to its high sugar content. Thus, it may be necessary to consume WJ slowly, over a number of hours [168].

### 6.7. Taurine

Taurine, a sulfur-containing amino acid produced endogenously from cysteine, is found abundantly in skeletal muscle. It has strong antioxidant activity, and several studies have associated its intake with cytoprotective actions against various tissue pathologies [169,170,171,172]. Conversely, taurine-depleted skeletal muscle has been associated with accelerated aging and incidence of muscular disorders [173,174]. 

In rats, taurine intake has been associated with an increased antioxidant status and decreased blood markers of oxidative stress and muscle fatigue following downhill running [175]. In humans, multiple studies have linked taurine supplementation with the attenuation of post-exercise blood markers of oxidative stress [176,177,178]. Da Silva et al. [178] showed that daily taurine supplementation for 3 weeks prior to eccentric elbow exercise reduced post-exercise muscle soreness, improved muscle force recovery and limited oxidative damage, despite no changes in antioxidant status or the inflammatory response. In addition, McLeay et al. [179] associated multi-day taurine supplementation in the post-exercise period (0.1 g/kg BW/day) with improved muscle force recovery at 72 h post-exercise but no difference in blood markers of muscle fatigue. 

Only one study has associated taurine intake with decreased inflammation, and this involved a multi-day muscle-fatiguing exercise protocol designed to simulate a soccer tournament [180]. However, no physical measures were assessed so the authors could not correlate these changes with improved muscle recovery.

In general, previous studies have employed taurine supplementation protocols of several weeks prior to exercise with doses ranging from 0.15 g/kg BW/day (approx. 1 g/day) to 6 g/day [175,176,177,180]. However, McLeay et al. [179] also showed an improved physical recovery when taurine was consumed twice daily for 72 h post-exercise (0.1 g/kg BW/day). Overall, taurine has the potential to impact post-exercise recovery, but further research is required to determine the most appropriate supplementation protocol. 

### 6.8. Bromelain and Other Proteases

Bromelain, found in a high concentration in developing pineapple stems, belongs to the protease family, a group of enzymes that function in the breakdown of proteins and play roles in food digestion, blood clotting and inflammation. Proteases have been proposed as potential regulators of the post-exercise inflammatory response [181].

In highly trained male road cyclists, supplementation with bromelain (1000 mg/day) during a 6-day cycling stage race reduced subjective feelings of fatigue and there was a trend for better maintenance of the testosterone concentration during the race, suggestive of improved recovery. Despite these findings, no changes in blood markers of fatigue were observed following supplementation [182]. Another study conducted in untrained males failed to show any effects of bromelain supplementation (900 mg/day for 4 days) on recovery following intense elbow exercise [183].

Bromelain may have a stronger effect on post-exercise recovery when combined with other proteases. Two studies found an association between multi-day consumption of a protease supplement containing 99.9 mg bromelain and improvements in muscle force recovery following downhill treadmill running [181,184], with one [184] also showing a decrease in post-exercise muscle soreness. In addition to this improved physical recovery, Buford et al. [181] recorded post-exercise decreases in markers of inflammation and fatigue, although no change in markers of muscle damage were found. 

Thus, there is some evidence that proteases may improve post-exercise recovery, although the mechanism by which this occurs is unknown. It appears that bromelain is more effective when ingested in small doses in combination with other proteases [17]. However, bromelain and other proteases can only act as anti-inflammatory agents when ingested separately from food [183].

## 7. Fatty Acids

### Fish Oils (Omega-3 Fatty Acids)

Fish oils, such as those from salmon, mackerel and tuna, are abundant in omega-3 polyunsaturated fatty acids (n-3 PUFA), primarily eicosapentaenoic acid (EPA) and docosahexaenoic acid (DHA) [185]. n-3 PUFA initially attracted research attention as populations with diets abundant in these compounds, such as the Greenland Inuit, have low incidences of cardiovascular disease [186]. Subsequently, the cardioprotective effects of n3-PUFA have been attributed to factors such as decreased inflammation, scavenging of RONS and positive changes in relation to the blood, including a drop in blood pressure. Based on these effects, it has been suggested that n3-PUFA may also have beneficial effects on post-exercise recovery [187,188,189].

n-3-PUFA, particularly EPA, exhibit their anti-inflammatory effects by becoming incorporated into the membranes of cells where they replace arachidonic acid, a compound associated with the generation of pro-inflammatory and oxidative compounds [188]. Accordingly, long-term supplementation with n3-PUFA (1800–3000 mg/day for 4–8 weeks) has been associated with a decrease in markers of oxidative stress [189] and/or inflammation [187,190,191] following various muscle-damaging eccentric exercise tasks. In terms of physical function, long-term n-3 PUFA supplementation may decrease post-exercise muscle soreness [187,190] and inflammation (measured by changes in limb girth) [190] and improve aspects of muscle function, such as peak power capacity [187] and range of motion [190,191]. However, data on force recovery are inconclusive. A study on upper body (elbow flexion) exercise noted an improvement following supplementation [191], while no change in this parameter was observed in two studies involving muscle-damaging leg exercise [187,190]. 

Although the effects of n-3 PUFA are generally attributed to cell membrane incorporation, which would require a long-term supplementation protocol, Jakeman [192] investigated whether a large acute dose of n-3 PUFA (1 g per 10 kg BW) given post-exercise could also aid in recovery. The effects of two n-3 PUFA supplements, containing high and low concentrations of EPA, on recovery following drop jump exercise were measured. While no changes in blood inflammatory and muscle damage markers or muscle soreness were found, the high-EPA group showed improvements in muscle performance tasks and force recovery. However, these effects were not observed in the low-EPA group.

Thus, in terms of dose recommendations, while daily long-term supplementation of at least 4 weeks has been the general practice for research on n-3 PUFA [187,189,190], it appears that positive effects on physical recovery may also occur with acute supplementation [192], a protocol that may invoke greater adherence. It is also important to note that when consumed excessively (>5 g/day) over a long period of time, fish oil consumption has been associated with increases in lipid peroxidation and oxidative stress, suppression of the inflammatory response and a decreased blood clotting ability [187,189]. Consequently, 100 IU of d-α-tocopherol/dl-α-tocopherol acetate is sometimes added to n-3 PUFA supplements to mitigate the occurrence of lipid peroxidation [190].

## 8. Vitamin D

The parathyroid hormone vitamin D is primarily synthesized endogenously in the skin as vitamin D3 following exposure to ultraviolet B radiation through sunlight [17,193]. Nutritionally, vitamin D can be consumed in foods such as oily fish, mushrooms and eggs as either vitamin D2 or D3, of which the D3 form is considered to have greater biological significance [193]. In the liver and kidneys, vitamin D undergoes further modifications that result in the formation of its biologically active form, calcitriol, which is a key regulator of calcium and phosphate homeostasis and is involved in the functioning of numerous tissues in the body, generally via the vitamin D receptor [193]. 

Given the importance of sun exposure to vitamin D generation in the body, vitamin D insufficiency (<30 nmol/L) is prevalent, particularly in individuals with mostly indoor lifestyles, during the winter months, and in athletes, who have higher vitamin D requirements. Notably, previous research has identified that the proportion of people meeting the daily recommended allowance for vitamin D from foods may be as low as 2% [194]. Previous research has shown a link between vitamin D insufficiency and greater post-exercise muscle damage [195] and inflammation [196], indicating that vitamin D supplementation may be important for these individuals.

Vitamin D has been proposed as a post-exercise recovery aid due to its positive effects on skeletal muscle regeneration through the promotion of muscle cell development and the immune response by helping to maintain appropriate levels of pro- and anti-inflammatory compounds [193]. However, its effectiveness in previous studies has differed depending on the type and dose given and the characteristics of the participants. Supplementation with 4000 IU vitamin D3 for 4–6 weeks improved muscle force recovery following muscle-damaging exercise in groups of untrained males with varied pre-supplementation vitamin D statuses [197,198]. In addition, increases in blood markers of muscle damage were attenuated in the first 24 h post-exercise but not thereafter [198]. Unfortunately, markers of inflammation were not measured, so it is unknown whether these effects were correlated with the immune response. Two further studies investigated the effects of vitamin D3 on post-exercise recovery in trained ultramarathon runners who were vitamin D-sufficient prior to supplementation. Following a muscle-damaging downhill running protocol, participants who consumed 2000 IU/day vitamin D3 for 3 weeks showed reductions in some blood markers of muscle damage (CK, MB) and inflammation (IL-6, TNF-ɑ) [199]. Conversely, markers of the inflammatory response did not differ following the completion of a 100 km ultramarathon following supplementation with a high dose of vitamin D3 (10,000 IU/day) for 2 weeks prior to exercise [200].

In contrast to the generally positive findings for vitamin D3, supplementation with vitamin D2 (sourced from Portobello mushrooms) has not been associated with physical or biochemical indicators of improved muscle recovery in vitamin D insufficient [201] or sufficient [63] individuals. Interestingly, following a 90 min eccentric exercise protocol, NASCAR pit crew athletes supplemented with 3800 IU/day vitamin D for 6 weeks had higher concentrations of markers of muscle damage than non-supplemented individuals, indicating an increased muscle damage response [63].

Thus, there is some evidence that vitamin D3 may be used as a supplement to enhance post-exercise recovery, particularly in the winter months when sunlight-induced endogenous synthesis is reduced. However, vitamin D2 does not appear to induce any positive effects associated with post-exercise recovery. In terms of a supplemental dose, positive results have been seen with quantities ranging from 2000 IU/day Vitamin D3 for 3 weeks to 4000 IU/day for 6 weeks [197,198,199]. A review of the literature in this area states that doses below 10,000 IU/day are not usually associated with toxicity, while those equal to or above 50,000 IU/day for several weeks or months may lead to hypercalcaemia, a condition associated with bone weakening [193].

## 9. Caffeine

Caffeine is one of the most well-established ergogenic aids, with significant evidence showing its beneficial effects for the performance of both resistance and endurance exercise-based tasks, particularly during states of mental fatigue or sleep deprivation [202,203]. Caffeine produces stimulatory effects via its antagonistic actions on adenosine, a component of the sympathetic nervous system that is associated with feelings of pain and fatigue. Thus, it has been suggested that caffeine intake could also reduce muscle soreness in the post-exercise period, potentially improving the ability to train and compete while recovering from muscle-fatiguing exercise [202,203]. In addition, caffeine-induced modulation of sympathetic nervous system activity may affect energy utilization, muscle contractile processes, hormone release and cognition. 

Hurley et al. [204] associated pre-exercise caffeine intake (5 mg/kg BW, 1 h pre-exercise) with reduced muscle soreness following multiple sets of bicep curl exercise and an improved performance and rating of perceived exertion in the later sets of the exercise. Using the same supplementation protocol, Duncan et al. [205] recorded a reduction in post-exercise muscle pain following bench press exercise to failure. In terms of energy use, Pederson et al. [206] showed a link between consumption of a high dose of caffeine (8 mg/kg) prior to cycling exercise to exhaustion with greater post-exercise muscle glycogen accumulation, suggestive of improved glycogen resynthesis. 

However, a series of studies by Machado and colleagues [207,208,209] found no effect of pre-exercise caffeine intake (4.5–5.5 mg/kg, 45 min pre-exercise) on markers of inflammation or muscle damage in professional and recreational soccer players. Further, Vimercatti [210] reported no differences in markers of muscle fatigue or inflammation following moderate treadmill running between groups supplemented with caffeine vs. a placebo 1 h prior to exercise.

Given that 75% of ingested caffeine is removed from the circulation within 6–7 h, to benefit post-exercise recovery [211,212], it may be necessary to consume a maintenance dose of caffeine during the recovery period. Accordingly, Caldwell et al. [213] implemented a multi-day supplementation protocol whereby caffeine (3 mg/kg BW) was ingested twice daily for 3.5 days following an endurance bike ride. This protocol successfully reduced muscle soreness during the recovery period and improved subjective scores of muscle function in relation to activities of daily living and exercise on the afternoon of the bike ride. Similarly, in low-caffeine-consuming females, Maridakis et al. [214] found that the consumption of caffeine in two doses (at 24 and 48 h) following electrically stimulated eccentric quadriceps exercise was associated with reduced muscle soreness and a small improvement in muscle force recovery. However, when using a protocol of this nature, it is necessary to consider the individual’s caffeine tolerance in relation to the timing of supplementation, as the consumption of caffeine in the afternoon may impair sleep, an essential component of the recovery process. 

## 10. Herbal Supplements

Herbal supplements include a wide range of plant-sourced compounds with numerous active components (phytonutrients). Many of these products have been used since ancient times to treat conditions relating to stress, inflammation and sleep [215,216]. In general, evidence regarding the effects of herbal supplements on post-exercise recovery is lacking and effects have not been measured directly. However, findings from sleep quality studies as well as investigations at the cellular level indicate that some of these compounds have the potential to enhance post-exercise recovery.

### 10.1. Ginger

Ginger (*Zingiber officinale*) was used traditionally in Indian (Ayurvedic) and Chinese medicine due to its anti-nausea and anti-inflammatory properties. Its key bioactive constituents are the gingerols, which give ginger its potent aroma. Ginger has been proposed to downregulate the inflammatory response, increase oxygen consumption and interrupt the processing of nociceptive (pain) signals, all of which may contribute to faster recovery from exercise [217,218].

In line with its proposed nociceptive effects, the consumption of ginger, either acutely or for up to 8 weeks, has been shown to reduce muscle soreness following muscle-damaging exercise [219,220,221,222]. However, despite decreased pain sensations, post-exercise muscle function does not appear to be influenced by ginger consumption [219,221,222]. The only study to record an improvement in post-exercise muscle function employed a high dose of ginger (4 g/day) for 5 days prior to eccentric arm exercise [223]. This attenuated the decline in muscle strength initially, but the effect did not persist, and was coupled with a later-than-normal peak in blood indicators of muscle damage and impaired flexibility. The authors suggested that ginger may have a limited window of anti-inflammatory function, so it may be necessary to continue supplementation in the post-exercise period.

In terms of anti-inflammatory actions, ginger does appear to reduce components of the immune response post-exercise when taken either acutely [219] or for 6–8 weeks prior to exercise [220,224]. A study that compared pre- and post-exercise ginger supplementation found that reductions in inflammatory compounds were only observed when ginger was consumed prior to exercise [219].

Thus, when consumed either pre- or post-exercise, ginger seems to produce anti-inflammatory and anti-muscle damage effects. The lowest dose shown to reduce post-exercise muscle soreness and inflammation is an acute dose of 2 g ginger powder (60 mg extract) [218,219], and quantities of up to 3 g/day for 8 weeks [220] have been used without adverse effects. However, at this stage, the anti-inflammatory and nociceptive functions of ginger do not seem to translate into physical improvements in muscle function, so ginger may not be an appropriate supplement for athletes wishing to enhance performance in subsequent training sessions or matches.

### 10.2. Ashwagandha

Ashwagandha (*Withania somnifera*) is a traditional Ayurvedan herbal supplement that is classified as an adaptogen—a supplement that promotes homeostasis and physical stability in a non-specific way rather than directly affecting factors related to negative health outcomes. It contains a variety of active components, including flavonoids, tannins, alkaloids, glycosides and steroid lactones (withanolides), of which the withanolides have been suggested to be the key factor associated with the positive effects of Ashwagandha [225].

Ashwagandha is a popular supplement in Southeast Asia and Southern Europe where it is believed to enhance general vitality, promote relaxation and reduce stress [226]. Among the conditions affected positively by supplementation are anxiety or mood disorders, muscle tremors and weakness, joint inflammation and rheumatic pain, and hypertension [13]. In terms of post-exercise recovery, there is evidence that long-term ashwagandha supplementation (300–600 mg taken daily for 8–12 weeks) may be associated with the optimization of sleep quality [227], a key factor in the promotion of adaptation to, and recovery from, physical effort [228]. In addition, ashwagandha may reduce the production of stress hormones associated with post-exercise muscle damage [227] and speed up the clearance of the muscle damage marker CK from the blood following resistance exercise [229]. Regarding physical recovery, when ashwagandha was consumed acutely (1000 mg) following muscle-damaging resistance exercise, non-significant increases in pain threshold and pain tolerance as well as a significant increase in peak power of the lower leg muscles were shown, indicative of improved recovery [13].

It appears that ashwagandha has the potential to enhance recovery, either by promoting relaxation or reducing the production of compounds associated with muscle damage. However, further research is required to determine whether physical improvements can be induced through the consumption of this herb.

### 10.3. Kava

Kava root extract (*Piper methysticum*) has been consumed since historic times by southern Pacific Islanders as a beverage with psychotropic, hypnotic and anti-anxiety effects [230]. Its active components are the kava lactones, a group of compounds with muscle relaxing, local anaesthetic, anti-anxiety and anti-convulsive properties when consumed in doses of ~140–250 mg/day [231]. The mechanism by which kava exerts its effects is unclear but may involve the neurotransmitters GABA, dopamine and norepinephrine, which are associated with mood [230]. 

However, while the muscle relaxant effects of kava suggest that it could improve post-exercise recovery when consumed in small doses, evidence of this effect is lacking. In addition, large doses of kava have been linked to unwanted side effects ranging from minor issues, such as drowsiness and lower back pain [230], to more extreme, although reversible, outcomes, including ataxia (a nervous system condition with alterations in movement and coordination), intoxication, sedation and paralysis of the extremities [231]. Long-term excess consumption has been associated with dry, scaly, yellowing skin and heart rate abnormalities, particularly among young athletes [232]. Of most concern is the potential for kava to interact with conventional medications and alcohol, leading to a risk of liver toxicity [230,232]. Thus, care must be taken with the dose size when consuming kava. 

### 10.4. Hops Extract

Hops extract, sourced from the hop cones, leaves, stems and rhizomes of the *Humulus lupulus* plant, is most well known as the bitter tasting component of beer [233]. Hops has also been used traditionally to treat pain, inflammation, anxiety and sleeping disorders and has been shown to have antibacterial, anti-cancer, anti-inflammatory, sedative and hypnotic properties. The active components in hops include numerous polyphenols and bitter acids, with the exact composition depending on the hop variety and climatic conditions during plant growth [234,235,236].

Little research on the effects of hops supplementation has been conducted in humans, although there is some evidence that the ingestion of ~500 mg of hops extract, either alone or in combination with valerian, may improve sleep quality or shorten the time taken to fall asleep [236], thereby having a potential positive influence on post-exercise recovery. In addition, evidence from cell-based studies shows the potential for hops to reduce inflammation. Recently, it was found that the treatment of macrophages (the cells from which inflammatory cytokines are released) with hops extract can suppress the expression of pro-inflammatory compounds associated with inflammation, such as IL-6, NO, and NF-KB [234]. 

However, caution must be taken with hops extract as it has been shown to have mild but distinct pro-estrogenic activity and, therefore, should not be consumed by women with a family history of breast cancer [237]. 

### 10.5. Valerian Root 

Valerian root, derived from the *Valeriana officinalis* plant, has a long history of use in Western Europe as a mild sedative and sleep aid and is currently employed to reduce stress and anxiety and to treat sleep disorders. Like the other herbs mentioned in this report, valerian’s mechanisms of action have not yet fully been determined. However, its numerous active constituents are predicted to affect the adenosine, GABA, and serotonin pathways, all of which are associated with mood and anxiety [233,238]. 

Like hops extract, potential effects of valerian on exercise recovery are related to its ability to improve sleep quality when taken alone or in combination with other herbs, of which hops extract and *Melissa officinalis* are the most common. In terms of dose, a recent review of the literature suggested that the consumption of 450–1410 mg of whole valerian root/rhizome for 4–8 weeks can effectively enhance sleep, while the effects of valerian extract appear to be more variable [238]. No previous studies have investigated the effects of valerian ingestion following exercise, so it is unknown whether any effects on sleep could enhance post-exercise recovery.

Although valerian has been demonstrated to be safe for human consumption, its sedative properties mean that it should not be consumed prior to driving or operating machinery and should not be taken in conjunction with alcohol or other sedatives, as it may exacerbate their effects. In addition, valerian should not be consumed by pregnant/lactating women [233].

### 10.6. Ginseng

Ginseng, sourced from several species of Araliaceae, is another herbal supplement with a long history of use for the enhancement of energy and well-being [239]. Its most commonly consumed form, *Panax ginseng* (red ginseng), originates from Korea and China and can be consumed in whole root, powder or extract forms. The primary active ingredients in ginseng are the saponins, particularly the ginsenosides, which vary in content and relative proportions between species [239]. Ginsenosides are thought to have positive effects on immune function, glucose metabolism, cognitive performance and oxidative stress [17], and supplementation with ginseng (2000 mg/day for 8 weeks) has been shown to significantly increase blood markers of antioxidant activity [240].

The consumption of ginseng has been associated with improved muscle force recovery following muscle-damaging exercise when taken acutely, in two doses (4000 mg pre- and post-exercise) [241] or for 8 days (100 mg/day) spanning the pre- and post-exercise periods [242], despite no concurrent change in blood markers of muscle damage [241,242]. There is also some evidence that acute (5000 mg) [243] or longer-term (1200 mg/day for 4 weeks) [244] supplementation with ginseng may reduce blood markers of muscular fatigue (lactate) following exercise. In terms of changes in inflammatory markers, the effect of ginseng is unclear and may depend on the supplementation protocol used. Thus, no conclusive remarks can be made in this regard.

Interestingly, a study that employed a ginseng supplement manufactured to contain highly bioavailable active components identified significantly greater maintenance of peak power output following a muscle-fatiguing exercise protocol, indicative of reduced muscle damage, but only in certain participants [245]. Thus, the effectiveness of ginseng as a recovery supplement may depend on whether the consumer is a “responder” or not.

## 11. Conclusions

In summary, muscle-fatiguing exercise invokes a biphasic response whereby skeletal muscle damage and metabolic perturbations initiate an inflammatory cascade that is necessary for muscle repair. Ultimately, this process contributes to adaptations that may enhance future exercise performance. However, symptoms of post-exercise muscle damage include pain and reduced muscle function which, depending on the level of damage incurred, can last for up to 14 days and can impact the ability of an athlete to train or compete during that period of time.

A wide range of nutritional compounds have been studied in regard to their ability to speed up post-exercise recovery. The majority of these have been investigated for their antioxidant characteristics with the remainder showing effects on sleep/relaxation or playing roles in critical bodily processes. Of the compounds included in this report, only two—tart cherry and omega-3 fatty acids—are supported by significant research evidence as having a high likelihood of having beneficial effects on improving post-exercise recovery, while a further five—BCAAs, HMB, creatine monohydrate, curcumin and pomegranate—appear to have beneficial effects but evidence of this is scarce (Table 1). For most compounds, evidence on their effectiveness is varied, and although a number of foods have shown potential to improve aspects of recovery, further research is required to determine the appropriate supplementation protocol and the population for whom the supplement may be effective [17]. Many of the compounds reviewed show potential in untrained populations, however it is less clear whether they will benefit well trained athletes for whom muscle damage will be less severe. As such, the development of new products, aimed at athletes and those who train regularly, should pay specific attention to the compounds shown to have an effect in trained populations. 

When adopting a supplementation protocol with any of the compounds mentioned in this review, it is important to consider the necessity of the post-exercise inflammatory response for adaptation to exercise. Although EIMD may reduce performance in the short term, there is significant evidence of the roles of inflammation and oxidative stress in activating signalling pathways associated with positive muscular adaptations, including mitochondrial biogenesis, muscle hypertrophy, and increased expression of the GLUT4 glucose transporter, allowing greater insulin responsiveness. Such changes ultimately improve the individual’s performance in the exercise modality that they are training [246,247]. However, research evidence suggests that there is a threshold beyond which muscle damage does not produce further adaptative benefits [248]. Thus, the general scientific consensus is that a periodized approach to nutritional supplementation, whereby these compounds are consumed during periods of high training load or tournament or stage race competitions, rather than on a regular basis, is most appropriate [17]. 

Overall, the potential for the use of foods and nutritional compounds to improve post-exercise recovery is promising, particularly in cases where an individual’s diet lacks components essential for protein synthesis (e.g., vegetarian, vitamin D deficient) and/or antioxidant activity (e.g., low in fruit/vegetables). Many of the compounds included in this review have been used since ancient times to treat conditions associated with inflammation and disease, and current evidence suggests that their potential for use in post-exercise recovery is also strong.

## Figures and Tables

**Table 1:** Effects of nutritional compounds on aspects related to post-exercise recovery.

Nutritional Compound/Food	Level of Evidence	Lowest Effective Dose	Key Findings
Vitamin C	Moderate	Initial dose of 1000 mg and then 400 mg/day for 12 days pre-exercise [25]	↑ Serum antioxidant status
			↓ Serum oxidative stress
			↓ Serum markers of muscle damage with multi-day protocols
Vitamin E	Low	250 mg, 1 h pre-exercise in hypoxic conditions [32]	↓ Serum markers of muscle damage and inflammation under hypoxic conditions
Vitamin C + Vitamin E	Low	N/A	No effect on muscle soreness or serum markers of muscle damage or antioxidant status
			Did not reduce adaptation to an endurance training programme
Tart Cherry	High	30 mL concentrate (containing ~600 mg polyphenols) consumed twice daily for at least 3 days pre-exercise [38,40,41,43]	↑ Muscle force recovery
			↑ Muscle function recovery
			↓ Post-exercise muscle soreness
			↓ Selected serum markers of inflammation
Pomegranate	Moderate/High	250 mL juice/30 mL concentrate (containing ~650 mg polyphenols) consumed twice daily for at least 5 days pre-exercise [53,54,55] or 1000 mg extract 30 min pre-exercise	↑ Force recovery in selected muscle groups
			↓ Systolic blood pressure, increased blood vessel diameter and blood flow
Blueberry	Moderate	200 mg (~420 mg polyphenols) consumed three times daily following exercise + 200 mg daily for the next 2 days [59]	Some evidence of improved muscle strength and function recovery
			No effect on blood markers of muscle damage/inflammation
Blackcurrant	Moderate	3.2 mg/kg (~80 mg anthocyanins) 1 h pre-exercise [65]	↑ Production of anti-inflammatory components of the immune response
Curcumin	Moderate/High	90 mg consumed 2 h pre-exercise [81]	↑ Muscle force and function recovery
			↓ Muscle soreness
			↓ Serum markers of muscle damage and oxidative stress
			↑ Serum antioxidant status
			↓ Selected inflammatory compounds in some studies
Beetroot juice	Moderate	150 mL (~385 mg polyphenols, 250 mg nitrate) consumed twice daily for 7 days (exercise on day 4) [98]	Varied results regarding muscle soreness, function and blood markers of muscle damage, oxidative stress and inflammation
Watermelon juice (L-citrulline	Moderate	500 mL (1.17 g L-citrulline) 1 h pre-exercise [110]	No effect on blood markers of muscle damage, fatigue or inflammation
			Variable effects on blood pressure
			Variable effects on muscle soreness and force recovery
Branched Chain Amino Acids	Moderate/High	80 mg/kg BW (~5.4 g) consumed 50 min pre-exercise [115]	↑ Improved muscle function recovery
			↓ Muscle soreness
			↓ Serum markers of muscle damage
			↓ Post-exercise serotonin concentration
β-Hydroxy β-methylbutyrate	Moderate/High	3 g 30 min pre- or post-exercise [132]	Long-term:
			↓ Markers of muscle protein breakdown/damage
			Some evidence of reduced inflammation and improved muscle force recovery
			Short-term/acute:
			Some evidence of reduced inflammation and muscle damage and improved muscle force recovery
Creatine monohydrate	Moderate/High	20 g/day (in 2–4 doses) for at least 5 days pre-exercise [150,151,154]	↓ Serum markers of muscle damage following exhaustive endurance exercise and, in some cases, resistance-based exercise
			↑ Post-exercise glucose response
			Some evidence of improved muscle force recovery following resistance-based exercise
l-Glutamine	Moderate	0.3 g/kg BW/day for 4 days prior to exercise [160]	↓ Muscle soreness in untrained individuals
			↓ Serum markers of muscle damage with a multi-day, high-dose supplementation protocol
			Some evidence of improved muscle force recovery in males
l-Carnitine	Moderate	2 g/day for 2 weeks prior to exercise [171]	↓ Muscle soreness
			↓ Serum markers of muscle damage
			Varied results regarding muscle function recovery
Taurine	Moderate	0.1 g/kg BW/day twice daily for 72 h post-exercise [183]	↓ Serum markers of oxidative stress
			↑ Improved muscle force recovery
			Little evidence for effects on blood markers of muscle fatigue and inflammation
Bromelain and other proteases	Moderate	Protease supplement containing 99.9 mg/day bromelain consumed for 24 days [185] or 900 mg/day (in 3 doses) bromelain consumed for 4 days prior to exercise [187]	Some evidence of reduced subjective fatigue and improved testosterone in highly trained cyclists when consumed during a stage race.
			↑ Improved muscle force recovery and reduced markers of inflammation when consumed as part of a protease supplement
Omega-3 fatty acids	High	1.8 g/day (324 mg EPA, 216 mg DHA) for 30 days pre-exercise [194] or 1 g (750 mg EPA, 50 g DHA) per 10 kg BW immediately post-exercise [196]	Long-term supplementation:
			↓ Serum markers of oxidative stress and inflammation and muscle soreness ↑Recovery of some aspects of muscle function (ROM, peak power) Varied evidence on muscle force recovery
			Acute large-dose, post-exercise supplementation: Some evidence of improved muscle function and force recovery
Vitamin D	Vitamin D3: ModerateVitamin D2: Low	2000 IU/day Vitamin D3 for 3 weeks pre-exercise [203]	Vitamin D3:
			↑ muscle force recovery with long-term supplementation
			Some evidence of attenuated serum markers of muscle fatigue and inflammation but this has not been shown consistently
			Vitamin D2: No evidence of improved recovery
Caffeine	Moderate	5 mg/kg BW 1 h prior to exercise [208,209]	↑ Post-exercise muscle glycogen accumulation
			Some evidence of reduced muscle soreness and improved muscle force recovery
			Greater benefit may be achieved with multiple maintenance doses post-exercise
Ginger	Low	2 g ginger powder (60 mg extract) consumed 30–60 min pre-exercise [222]	Not likely to affect post-exercise muscle function recovery
			↓ Selected serum markers of inflammation and muscle damage
Ashwagandha	Moderate	750 mg consumed immediately post-exercise [13]	Some evidence of improved sleep quality and a reduction in the production of stress hormones
			Some evidence of improved muscle function recovery and reduced muscle damage post-exercise.
Kava	Low	N/A	Little evidence of enhanced post-exercise recovery
Hops extract	Low	N/A	Little evidence of enhanced post-exercise recovery
Valerian root	Low	450 mg [242]	↑ Sleep quality
			Unknown if its effects would benefit post-exercise recovery
Ginseng	Moderate	100 mg/day for 8 days with exercise on day 5 [246]	↑ Muscle force recoveryT
			Some evidence of reduced lactate post-exercise
			Effect on blood markers of damage and inflammation is unclear

Level of evidence: High—Likely to have some benefits; moderate/high—appears to have beneficial effects but further research is required; moderate—results are varied and further research is required; low—little evidence to support its use.

## Data Availability

Not applicable.
